# Supplementary material for: Occurrence of virulence genes in multidrug-resistant Escherichia coli isolates from humans, animals, and the environment: One health perspective
Source: PLoS One. 2025 Jan 24;20(1):e0317874. doi: 10.1371/journal.pone.0317874 (PMC11760637; doi:10.1371/journal.pone.0317874)
Supplement: S1 Table — (DOCX) [file pone.0317874.s001.docx]

| **Target gene** | **Primers** | **Primer Sequences (5’----3’)** | **Annealing temp (°C)** | **Amplicon size (bp)** | **References** |
| --- | --- | --- | --- | --- | --- |
| *ompA* | *ompA-F* | AGCTATCGCGATTGCAGTG | 52 | 919 | [5] |
|  | *ompA-R* | GGTGTTGCCAGTAACCGG |  |  |  |
| *stx1* | *stx1-F* | \| TCTCAGTGGGCGTTCTTATG \| \| --- \| | 58 | 388 | [5] |
|  | *stx1-R* | \| TACCCCCTCAACTGCTAATA \| \| --- \| |  |  |  |
| *Bfp* | *bfp-F* | \| AATGGTGCTTGCGCTTGCTGC \| \| --- \| | 56 | 324 | [5] |
|  | *bfp-R* | \| GCCGCTTTATCCAACCTGGTA \| \| --- \| |  |  |  |
| *traT* | *traT-F* | GGTGTGGTGCGATGAGCACAG | 63 | 290 | [5] |
|  | *traT-R* | CACGGTTCAGCCATCCCTGAG |  |  |  |
| *eaeA* | *eaeA-F* | \| ATGCTTAGTGCTGGTTTAGG \| \| --- \| | 58 | 248 | [5] |
|  | *eaeA-R* | \| GCCTTCATCATTTCGCTTTC \| \| --- \| |  |  |  |
