# Supplementary material for: Occurrence of virulence genes in multidrug-resistant Escherichia coli isolates from humans, animals, and the environment: One health perspective
Source: PLoS One. 2025 Jan 24;20(1):e0317874. doi: 10.1371/journal.pone.0317874 (PMC11760637; doi:10.1371/journal.pone.0317874)
Supplement: S2 Table — (DOCX) [file pone.0317874.s002.docx]

| \| **Program** \| \| --- \| | **Targeted Genes** | **Amplification conditions for virulence genes** | | | | | |
| --- | --- | --- | --- | --- | --- | --- | --- | --- |
|  |  | **Initial Denaturation** | **Denaturation** | **Annealing** | **Primer extension** | **Final extension** | **Number of cycles** |
| PCR 1 | *ompA,*  *traT* | \| 94°C for 5min \| \| --- \| | \| 94°C for 1min \| \| --- \| | \| 58°C for 30sec \| \| --- \| | \| 68°C for 3min \| \| --- \| | \| 72°C for 10min \| \| --- \| | \| x30 \| \| --- \| |
| PCR 2 | *stx1,*  *eae* | \| 95°C for 5min \| \| --- \| | \| 95°C for 30sec \| \| --- \| | \| 55°C for 30sec \| \| --- \| | \| 72°C for 3min \| \| --- \| | \| 72°C for 7min \| \| --- \| | \| x35 \| \| --- \| |
| PCR 3 | *Bfp* | \| 95°C for 5min \| \| --- \| | \| 95°C for 30sec \| \| --- \| | \| 55°C for 30sec \| \| --- \| | \| 72°C for 3min \| \| --- \| | \| 72°C for 7min \| \| --- \| | \| x35 \| \| --- \| |
