# Supplementary material for: Occurrence of virulence genes in multidrug-resistant Escherichia coli isolates from humans, animals, and the environment: One health perspective
Source: PLoS One. 2025 Jan 24;20(1):e0317874. doi: 10.1371/journal.pone.0317874 (PMC11760637; doi:10.1371/journal.pone.0317874)
Supplement: S3 Table — (DOCX) [file pone.0317874.s003.docx]

| **Genes** | **Different Sample Sources n (%)** | | | | **Total (n=50)** | **p-value** |
| --- | --- | --- | --- | --- | --- | --- |
|  | **Human (n=15)** | **Poultry (n= 14)** | **Pigs (n=15)** | **River water (n=6)** |  |  |
| *ompA* | 12 (80%) | 10 (71.4%) | 10 (66.7%) | 4 (66.7%) | 36 (72%) | 0.855 |
| *traT* | 14 (93.3%) | 11 (78.6%) | 13 (86.7%) | 3 (50%) | 41 (82%) | 0.122 |
| *eaeA* | 13 (86.7%) | 13 (92.9%) | 7 (46.7%) | 6 (100%) | 39 (78%) | 0.005 |
| *Bfp* | 14 (93.3%) | 9 (64.3%) | 13 (86.7%) | 5 (83.3%) | 41 (82%) | 0.062 |
