# Supplementary material for: Occurrence of virulence genes in multidrug-resistant Escherichia coli isolates from humans, animals, and the environment: One health perspective
Source: PLoS One. 2025 Jan 24;20(1):e0317874. doi: 10.1371/journal.pone.0317874 (PMC11760637; doi:10.1371/journal.pone.0317874)
Supplement: S4 Table — (DOCX) [file pone.0317874.s004.docx]

| **Virulence Genes** | **Different Sample Sources n (%)** | | | | **Total**  **n=50**  **(%)** |
| --- | --- | --- | --- | --- | --- |
|  | **Humans n=15(%)** | **Poultry n=14(%)** | **Pig n=15**  **(%)** | **River Water n=6**  **(%)** |  |
| *ompA* | 0 (0) | 0 (0) | 1 (6.7) | 0 (0) | 1 (2) |
| *traT* | 0 (0) | 0 (0) | 1 (6.7) | 0 (0) | 1 (2) |
| *ompA+bfp* | 0 (0) | 0 (0) | 1 (6.7) | 0 (0) | 1 (2) |
| *traT+bfp* | 2 (13.3) | 0 (0) | 1 (6.7) | 0 (0) | 3 (6) |
| *traT+eaeA* | 0 (0) | 1 (7) | 2 (13.3) | 0 (0) | 3 (6) |
| *ompA+eaeA* | 0 (0) | 0 (0) | 0 (0) | 1 (16.7) | 1 (2) |
| *bfp+traT+eaeA* | 1 (6.7) | 3 (21) | 2 (13.3) | 2 (33.3) | 8 (16) |
| *ompA+bfp+eaeA* | 1 (6.7) | 4 (29) | 0 (0) | 2 (33.3) | 7 (14) |
| *ompA+traT+bfp* | 0 (0) | 0 (0) | 4 (26.6) | 0 (0) | 4 (8) |
| *ompA+traT+eaeA* | 1 (6.7) | 0 (0) | 1 (6.7) | 0 (0) | 2 (4) |
| *ompA+traT+bfp+eaeA* | 10 (66.6) | 6 (43) | 2 (13.3) | 1 (16.7) | 19 (38) |
