# Supplementary material for: Occurrence of virulence genes in multidrug-resistant Escherichia coli isolates from humans, animals, and the environment: One health perspective
Source: PLoS One. 2025 Jan 24;20(1):e0317874. doi: 10.1371/journal.pone.0317874 (PMC11760637; doi:10.1371/journal.pone.0317874)
Supplement: S5 Table — (DOCX) [file pone.0317874.s005.docx]

| **AMR Genes** | **Virulence Genes** | | | |
| --- | --- | --- | --- | --- |
|  | **Correlation Coefficients (r)** | | | |
|  | ***ompA*** | ***traT*** | ***eaeA*** | ***Bfp*** |
| *bla-CTX-M* | -0.08 | -0.21 | 0.23 | -0.3 |
| *bla-TEM* | 0.39 | -0.21 | 0.23 | 0.27 |
| *qnrB* | -0.11 | 0.18 | -0.33 | -0.26 |
| *qnrS* | -0.35 | 0.25 | 0.24 | 0.12 |
