# Supplementary material for: Occurrence of virulence genes in multidrug-resistant Escherichia coli isolates from humans, animals, and the environment: One health perspective
Source: PLoS One. 2025 Jan 24;20(1):e0317874. doi: 10.1371/journal.pone.0317874 (PMC11760637; doi:10.1371/journal.pone.0317874)
Supplement: S1 Raw images — (PDF) [file pone.0317874.s006.pdf]

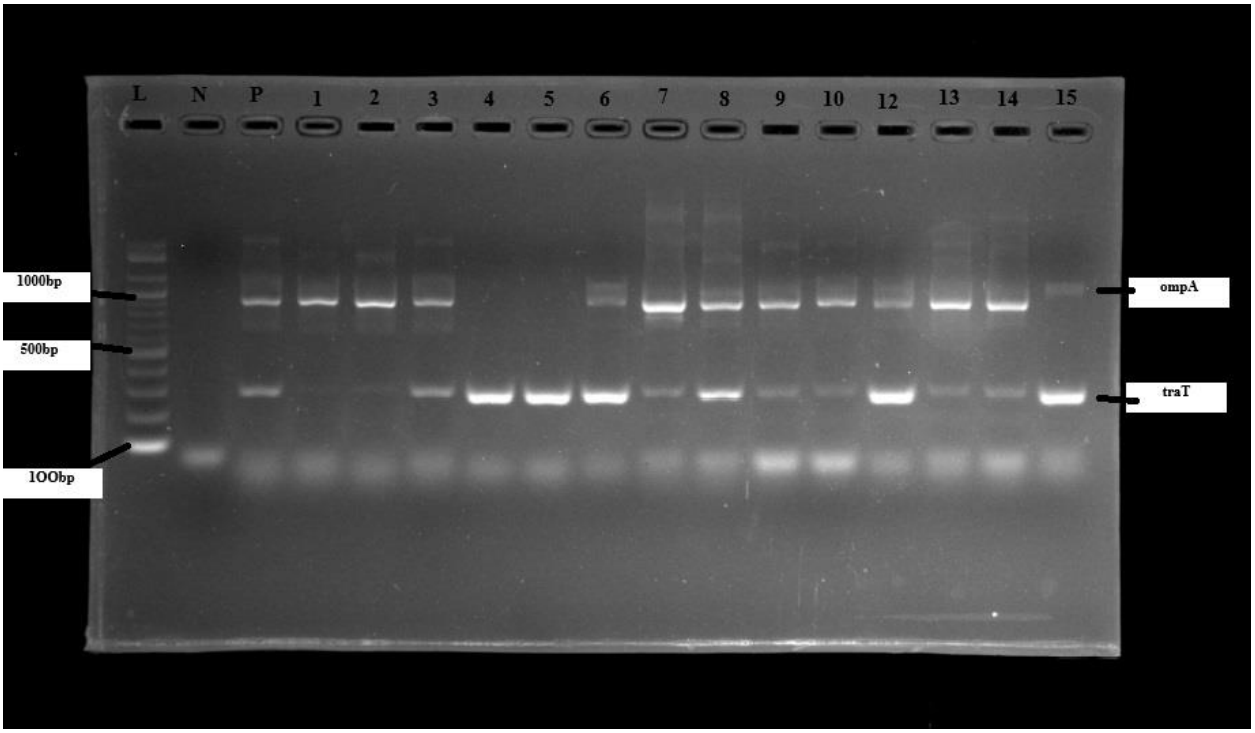

Fig. 2 Gel electrophoretic bands of virulence genes (*ompA* and *traT*).

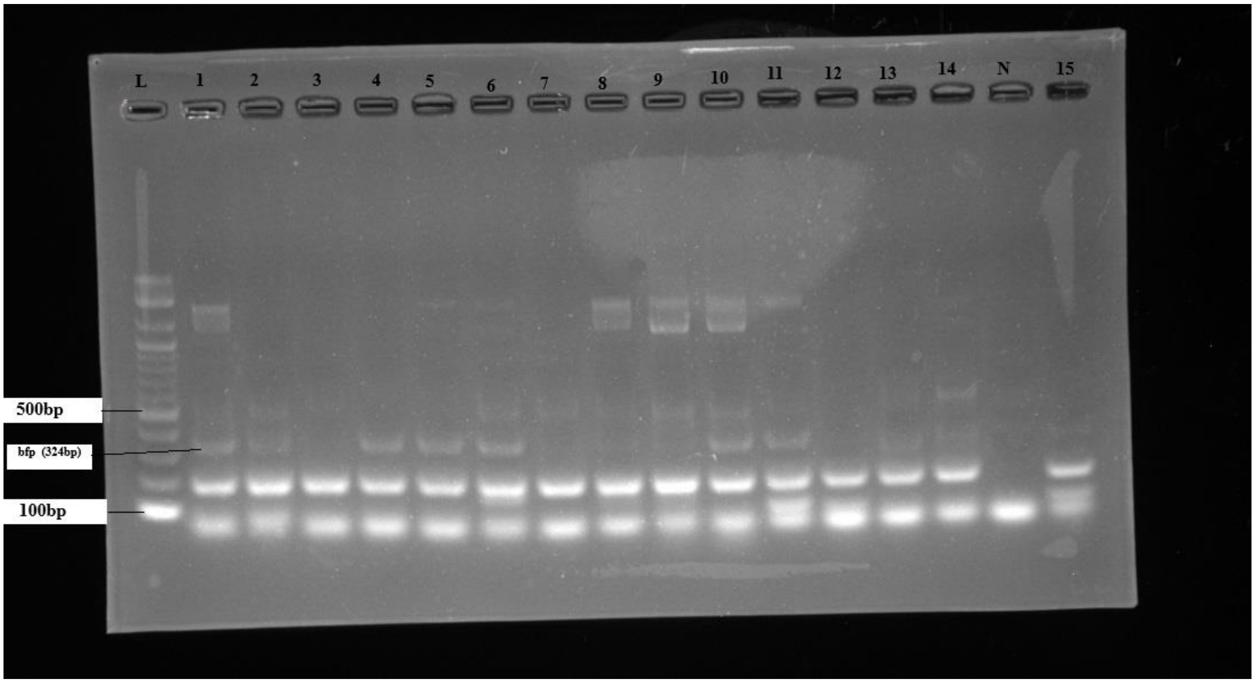

Fig. 4 Gel electrophoretic bands of virulence gene *bfp*.
