# Supplementary material for: Occurrence of virulence genes in multidrug-resistant Escherichia coli isolates from humans, animals, and the environment: One health perspective
Source: PLoS One. 2025 Jan 24;20(1):e0317874. doi: 10.1371/journal.pone.0317874 (PMC11760637; doi:10.1371/journal.pone.0317874)
Supplement: Attachment — Submitted filename: PONE-D-24-37013.pdf [file pone.0317874.s007.pdf]

Manuscript Number: PONE-D-24-37013

Title: Occurrence of virulence genes *bfp, ompA, traT, eaeA, and stx1* in multidrug-resistant Escherichia coli isolates from humans, animals, and the environment: One Health perspective

Overview and general recommendation:

This subject is important because the current study detected prominent virulence genes *bfp, ompA, traT, eaeA and stx1* in MDR E. coli isolates from humans, animals, and the environment and to determine their association with ESBL genes (*blaCTX-M, blaTEM, and blaSHV*) and plasmid-mediated quinolone resistance (PMQR) genes (*qnrA, qnrB, qnrS, qnrC, qnrD, qepA, and aac (6)-Ib-cr*).

I found the manuscript well written, well described and the references were new. I listed minor points below for editing.

Comments to Author:

Minor points

1- In Background; page3, 49 line: You wrote virulence genes **(VRs)!!!** That's incorrect abbreviation; kindly correct it to **(VGs)**.

2- In Background; page3, 53 line: you wrote **blaOXA-4!!!** That's incorrect; kindly correct it to **blaOXA-48**

3- In Background; page4, 81 line and 85 line: The rule of manuscript writing is to avoid using (We). So you should delete (**We**) and use formal scientific words (**This study or The current study or The present study**).

4- In Biochemical identification of the isolates; page5, 105 line: you wrote **Isolates were identified as described previously [4,26,29,31]. Briefly**…..!!! Kindly delete this sentence and directly write: **The isolates were identified by colonial morphology, Gram stain**…. Do not repeat the sentences because this makes your manuscript weak.

5- In DNA extraction for the screening of virulence genes; page6, 124 line: you wrote whole the practical method but **without writing any references!!!** Kindly mention the references that you dependent on them.

6- In PCR mixture for the detection of virulence genes; page7, 136 line: The rule of manuscript writing is to avoid using (We). So you should delete (**We**) and use formal scientific words (**This study or The current study or The present study**).

7- In Visualization of PCR products by electrophoresis; page7, 154 line: you wrote whole the practical method but **without writing any references again!!!** Kindly mention the references that you dependent on them.

8- In discussion; page16, 268 line, 270,283, 287, 311, 315, 321, 325, 351 and 353line: The rule of manuscript writing is to avoid using (Our) and (We). So you should delete **(Our)** and (**We**) and use **formal scientific words** (**This study or The current study or The present study**).

9- In discussion; page16, 274 line: you wrote **the occurrence of 274 virulence gene stx1 was low, being 4% in Karatu and 0% in this study!!!** Why? You did not suggest any reason for it!!! **Try to mention accurate scientific reason.**

10- In conclusion; page20, 359 line, 361 and 363line: The rule of manuscript writing is to avoid using (We). So you should delete (**We**) and use **formal scientific words** (**This study or The current study or The present study**).

11- In conclusion; page20: **You should shortly mention the fact that you explained about the PCA** because this make your research stronger scientifically; you wrote it in discussion only "PCA showed that isolates from pigs had a higher proportion of virulence genes, followed by those isolated from the environment, with those from poultry and humans having the lowest proportion, which indicates their respective potential as a reservoir for the genes".

Best regards
