# Supplementary material for: Occurrence of virulence genes in multidrug-resistant Escherichia coli isolates from humans, animals, and the environment: One health perspective
Source: PLoS One. 2025 Jan 24;20(1):e0317874. doi: 10.1371/journal.pone.0317874 (PMC11760637; doi:10.1371/journal.pone.0317874)
Supplement: Attachment — Submitted filename: Response to review Letter 26 december.docx [file pone.0317874.s008.docx]

Date: 26^th^ December, 2024

Chief Editor,

PLOS ONE Journal

Dear Sir/Madam,

**RE: RESPONSE TO REVIEWERS COMMENTS REGARDING THE MANUSCRIPT TITLED “OCCURRENCE OF VIRULENCE GENES IN MULTIDRUG-RESISTANT *Escherichia coli* ISOLATES FROM HUMANS, ANIMALS, AND THE ENVIRONMENT: ONE HEALTH PERSPECTIVE**”

We sincerely appreciate the time and effort you and the reviewers have dedicated to evaluating our manuscript titled “**Occurrence of virulence genes in multidrug-resistant *Escherichia coli* isolates from humans, animals, and the environment: One Health Perspective**”. We are grateful for the constructive feedback provided, which has helped us improve the quality and clarity of our work. Please, find below a point-by-point response to the comments raised by the reviewers and the academic editor.

**Reviewer #1 Comments:**

**1. Comment:**	The names of resistance genes are not necessarily to be described in the title. It could be "Occurrence of virulence genes among multidrug-resistant *Escherichia coli* isolates from humans, animals, and the environment: A One Health Perspective"

**Response:**	**The names of resistance genes have been removed in the title as suggested (Page 1, line 1)**

**2. Comment:**	There is lots of grammar or English mistakes in the article

**Response:**	**The manuscript has been checked using Quillbot, an online tool to remove grammatical errors and improve language**

**3. Comment:**	Novelty statement or the need of project should be more focused, need revision of Line 81. Here describe more objective of the study

**Response**:	**In addition to the originally stated objective, the statement saying; “The influence of resistance genes on the occurrence of different virulence genes was demonstrated using principal component analysis (PCA). To find out if isolates from humans, pigs, poultry, or river water had more virulence genes, PCA ellipses were employed. This study was able to assess their respective capability as gene reservoirs as a result” has been added. Page 5, Line 86 to 92**

**4. Comment:**	More references should be added from other parts of the world including Veterinary settings, to provide strong One Health Perspective and Global Concern.

**Response:	The references have been added as requested. Please refer to references number 35, 37 and 38**

**5. Comment:**	Line 90-92, this is not required to describe the link.

**Response:**	**The link has been removed as suggested (Page 5, line 99 to line 102)**

**6. Comment:**	Line 102-103: Dates are not required to be mentioned

**Response:**	**Dates have been omitted as suggested (page 5, line 105 – 109)**

**7. Comment:**	Tables are good, however, it is suggested to add at least one figure using data of Table 3

**Response:**	**Figure number 1 has been added to represents data of table 3**

**8. Comment:**	Discussion section is more focused on results. Some of the critical points should be added.

**Response**:	**Modifications have been done throughout the discussion sections as per suggestion**

**Additional Revisions:** In addition to addressing the reviewers' comments, we have made minor edits throughout the manuscript to improve clarity and resolve typographical errors. These changes are detailed in the tracked-changes version of the manuscript for your reference.

We hope that our revisions meet the expectations of the reviewers and the academic editor and we look forward to your feedback.

Thank you once again for considering our work for publication in PLOS ONE.

Sincerely,

Elieshiupendo Niccodem

**Corresponding author**
